# Supplementary material for: HIVprotI: an integrated web based platform for prediction and design of HIV proteins inhibitors
Source: J Cheminform. 2018 Mar 9;10:12. doi: 10.1186/s13321-018-0266-y (PMC5845081; doi:10.1186/s13321-018-0266-y)
Supplement: Supplementary file 2 — Additional file 2. Source code of HIVProtI web server. [file 13321_2018_266_MOESM2_ESM.zip › HIVprotI_Source-code/hivprotI/weka-3-6-6/doc/weka/gui/beans/Startable.html]

Startable


---


|  |  |  |
| --- | --- | --- |
| **Overview** \| **Package** \| **Class** \| **Tree** \| **Deprecated** \| **Index** \| **Help** \| **Weka's home** | |  |
| **PREV CLASS**   **NEXT CLASS** | **FRAMES**    **NO FRAMES**     **All Classes** |  |
| SUMMARY: NESTED \| FIELD \| CONSTR \| METHOD | DETAIL: FIELD \| CONSTR \| METHOD |  |


---


## weka.gui.beans Interface Startable

**All Known Implementing Classes:**: Loader

---

``` public interface Startable ```

Interface to something that is a start point for a flow and
can be launched programatically.

**Since:**
:   1.0

**Version:**
:   $Revision: 6017 $

**Author:**
:   Mark Hall (mhall{[at]}pentaho{[dot]}org)

---

| **Method Summary** | |
| --- | --- |
| `java.lang.String` | `getStartMessage()`             Gets a string that describes the start action. |
| `void` | `start()`             Start the flow running |

**Method Detail**

### start

```
void start()
           throws java.lang.Exception
```

:   Start the flow running

    :   **Throws:**: `java.lang.Exception` - if something goes wrong

---


### getStartMessage

```
java.lang.String getStartMessage()
```

:   Gets a string that describes the start action. The
    KnowledgeFlow uses this in the popup contextual menu
    for the component. The string can be proceeded by
    a '$' character to indicate that the component can't
    be started at present.

    :   **Returns:**: a string describing the start action.


---


|  |  |  |
| --- | --- | --- |
| **Overview** \| **Package** \| **Class** \| **Tree** \| **Deprecated** \| **Index** \| **Help** \| **Weka's home** | |  |
| **PREV CLASS**   **NEXT CLASS** | **FRAMES**    **NO FRAMES**     **All Classes** |  |
| SUMMARY: NESTED \| FIELD \| CONSTR \| METHOD | DETAIL: FIELD \| CONSTR \| METHOD |  |


---
